# Modulatory Role of Nitric Oxide/cGMP System in Endothelin-1-Induced Signaling Responses in Vascular Smooth Muscle Cells

**DOI:** 10.2174/157340310793566055

**Published:** 2010-11

**Authors:** Georgia Kapakos, Ali Bouallegue, Grace Bou Daou, Ashok K Srivastava

**Affiliations:** Laboratory of Cell Signaling, Montreal Diabetes Research Centre, Centre de Recherche du Centre Hospitalier de l’Université de Montréal (CRCHUM) – Technopole Angus and Department of Medicine, University of Montreal, Montreal, Quebec, Canada

**Keywords:** Endothelin-1, Nitric oxide, ERK1/2, PKB, VSMC, Proliferation, Hypertrophy, Vasculature.

## Abstract

Nitric oxide (NO) is an important vasoprotective molecule that serves not only as a vasodilator but also exerts antihypertrophic and antiproliferative effects in vascular smooth muscle cells (VSMC). The precise mechanism by which the antihypertrophic and antiproliferative responses of NO are mediated remains obscure. However, recent studies have suggested that one of the mechanisms by which this may be achieved includes the attenuation of signal transduction pathways responsible for inducing the hypertrophic and proliferative program in VSMC. Endothelin-1 is a powerful vasoconstrictor peptide with mitogenic and growth stimulatory properties and exerts its effects by activating multiple signaling pathways which include ERK 1/2, PKB and Rho-ROCK. Both cGMP-dependent and independent events have been reported to mediate the effect of NO on these pathways leading to its vasoprotective response. This review briefly summarizes some key studies on the modulatory effect of NO on these signaling pathways and discusses the possible role of cGMP system in this process.

## INTRODUCTION

Increased vascular smooth muscle cell (VSMC) hypertrophy, migration and proliferation are among key events that contribute to remodeling of the vasculature associated with cardiovascular diseases. In recent years, an important role of endothelin-1 (ET-1) in vascular hypertrophy and proliferation, leading to vasculopathies including atherosclerosis and hypertension has been suggested [1,2]. Conversely, nitric oxide (NO), originally described as a non-prostaglandin, endothelium-derived relaxing factor (EDRF), in addition to its potent vasodilator action, has emerged as an important vasculo-protective agent through its ability to exert anti-hypertrophic, anti-proliferative and anti-apoptotic responses in the cardiovascular system [3-7]. The balance between these two endothelium-derived opposing agents helps regulate vascular homeostasis. Even though previous studies have suggested possible interactions between NO and ET-1, the mechanistic contribution of NO on ET-1-induced vascular remodeling still remains poorly understood. The aim of this review is to highlight the effect of the NO system on the key signaling pathways induced by ET-1 that are linked to hypertrophy and proliferation of VSMC. 

## ET-1-INDUCED SIGNALING PATHWAYS

ET-1 is a potent vasoconstrictor peptide which also exhibits mitogenic [8-11], and hypertrophic properties [12]. ET-1 has been shown to stimulate VSMC proliferation [13], migration [14], contraction [15], extracellular matrix deposition remodeling and deposition [16-18], secretion of growth factors and inflammatory mediators [19]. It exerts its biological actions in a paracrine/autocrine fashion through the activation of two receptor subtypes, ET_A_ and ET_B _[20,21]. Both receptors belong to a large family of seven transmembrane guanine nucleotide-binding protein-coupled receptors (GPCRs). Each receptor couples to different G protein families. ET_A_ receptors are highly expressed in VSMC but are also found in cardiomyocytes, fibroblasts, hepatocytes, adipocytes, osteoblasts as well as in brain neurons [20,22]. ET_B_ receptors are predominantly expressed in endothelial cells, however, a relatively low level of expression in other cells including cardiomyocytes, hepatocytes, fibroblasts, osteoblasts, and VSMC has been reported [21]. These two ET-1 receptors exhibit somewhat different physiological roles [22-25]. ET-1 induced activation of ET_A_ receptors on smooth muscle cells is believed to contribute to vasoconstriction, cell growth and cell adhesion, whereas binding of ET_B_ receptors leads to vasodilation through the release of NO and prostacyclin [3,25]. 

**Fig. (1):**
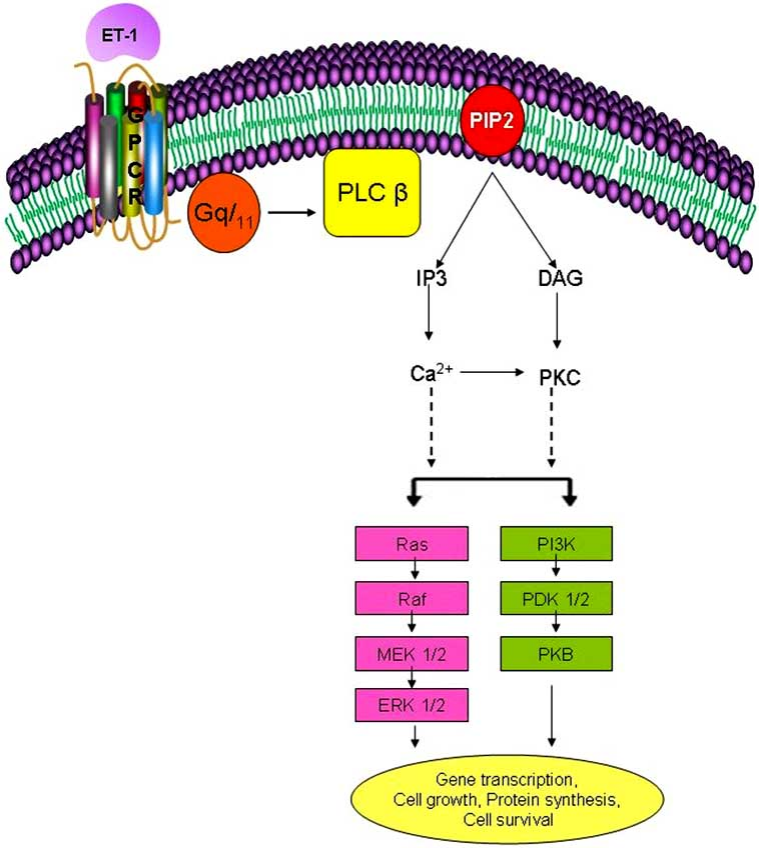
**ET-1-induced signaling cascade in VSMC.** ET-1 receptor stimulation leads to both Gqα, as well as βγ activation, which then activates PLC β. PLC β converts PIP2 to IP3 and DAG. IP3 is responsible for elevating intracellular calcium concentrations. DAG activates PKC. Through the activation of several downstream intermediates, Ca^2+^ alone or in partnership with PKC or other intermediates triggers the activation of Ras/Raf/MEK/ERK1/2 as well as PI3-K/PKB pathways. Activation of ERK and PKB signaling cascades play a key role in mediating various cellular responses such as gene transcription, protein synthesis, cell growth and cell survival.

The activation of the ET_A_ receptor triggers multiple signaling pathways (Fig. **1**) [26,27]. One of the primary targets include phosphoinositide-specific phospholipase C β (PLC β) [28,29], which hydrolyzes the membrane phospholipid phosphatidylinositol-4’-5’-biphosphate (PIP_2_) to generate two second messengers: hydrophobic diacylglycerol (DAG), and soluble inositol-1’,4’,5’-triphosphate (IP_3_). IP_3_ contributes to Ca^2+^ release from intracellular stores which play an important role in regulating the contractile response of the cell. DAG, together with Ca^2+^, activates the phosphatidylserine-dependent protein kinase, protein kinase C (PKC). PKC is a serine/threonine kinase that translocates from the cytosol to the cell membrane where it becomes activated and phophorylates several proteins [30]. ET-1-induced activation of PKC in VSMC leads to protein synthesis [31], cellular proliferation [30,32] and contraction [33]. Downstream of PKC, ET_A_ receptor activation also results in the stimulation of the mitogen activated protein kinases (MAPKs), a family of serine/threonine protein kinases known to promote cell growth [34]. These MAPK include ERK1/2, c-Jun-NH2-terminal kinase (JNK) and p38MAPK [27,35-38]. ET-1 has also been shown to activate the phosphatidylinositol-3-kinase (PI3K)/ protein kinase B (PKB) pathway [27,39,40], which signals cell growth, transformation, differentiation, motility and survival [41,42]. 

## NITRIC OXIDE SYSTEM

NO is a short-lived free radical generated by the oxidation of L-arginine to L-citrulline by a reaction catalyzed by nitric oxide synthase (NOS). Three distinct NOS enzymes have been identified, each a product of a unique gene. These are classified as neuronal NOS (nNOS or NOS-1), inducible NOS (iNOS or NOS-2) and endothelial NOS (eNOS or NOS-3) [43]. It was originally believed that NOS-1 and NOS-3 are constitutively expressed in neuronal and endothelial tissues, whereas NOS-2 expression is induced in response to cytokines or endotoxin activation. However, later studies have demonstrated a constitutive expression of NOS-2 in some tissues [44,45], and evidence has been presented to indicate that NOS-1 and NOS-3 may also be regulated by certain stimuli [46-50]. The principal mechanism by which NO exerts its biological effects involves the activation of soluble guanylate cyclase (sGC), a heterodimeric NO receptor that becomes activated once NO binds to its heme containing group. Binding of NO leads to allosteric modification of sGC, resulting in enhanced catalytic activity [51]. GCs are enzymes that catalyze the conversion of intracellular GTP into the second messenger cyclic guanosine 3’5’-monophosphate (cGMP) [52]. Thus formed, cGMP binds and activates cGMP-dependent protein kinase (PKGs) [52]. Two different types of PKG, type I (PKG-1) and type II (PKG-2) are expressed in mammalian tissues, however, their relative distribution is tissue and species dependent [53,54]. In cardiovascular tissues, a predominant expression of PKG-1 has been reported, and its role in mediating the anti-proliferatve effect of cGMP has been suggested [54-57]. PKG-1 elicits its effects through serine/threonine phosphorylation of multiple substrates which include IP_3_ receptor, phospholamban, troponin, the myosin light chain phosphatase, c-raf kinase, Ca^2+^ and K^+^ channels [52, 58-62]. NO can also exert its effects through non sGC/cGMP/PKG-dependent mechanisms, which include changes in cAMP signaling [63] or through the production of highly reactive peroxynitrite radical, capable of post-translational modification of protein function by nitration of tyrosine residues [64-66]. 

## NO MODULATION ON ET-1 SIGNALING

**Fig. (2):**
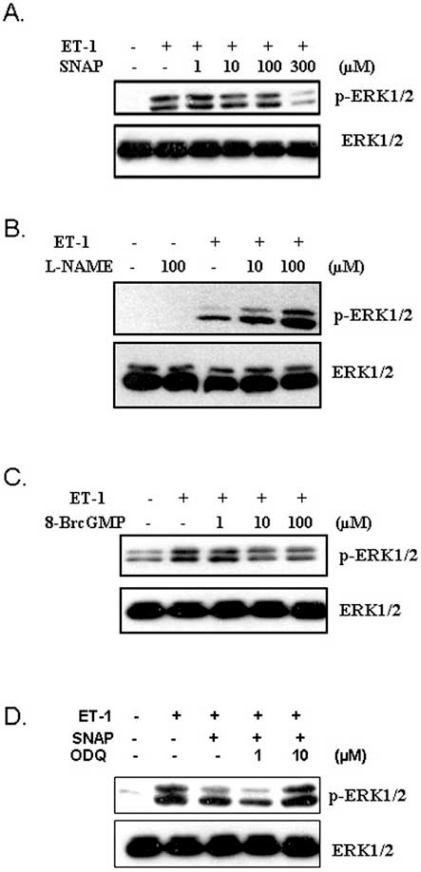
**Modulation of ET-1-induced ERK1/2 phosphorylation by NO/cGMP system in A10 VSMC.** **A.** The NO donor SNAP dose-dependently reduced ET-1-induced ERK1/2 phosphorylation. **B**.L-NAME, NO synthase inhibitor, dose-dependently potentiated ET-1-induced ERK1/2 phosphorylation. **C**. The cGMP analog, 8- Br-cGMP, mimicked SNAP effect by dose-dependently attenuating ET-1-induced phosphorylation of ERK1/2. **D**.ODQ, an inhibitor of sGC, reversed SNAP-induced attenuation of ERK1/2 phosphorylation in response to ET-1. Adapted from [76].

**Fig. (3):**
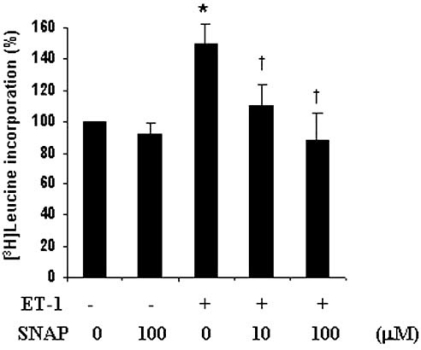
**Attenuation of ET-1-induced total protein synthesis by SNAP in A10 VSMC**. NO donor SNAP dose-dependently inhibited ET-1-induced [^3^H] Leucine incorporation into total cellular protein. Adapted from [76].

Existence of a cross-talk between NO and ET-1 system within the cardiovascular system has been known for a long time [3]. It has been demonstrated that NO counteracts the vasoconstrictor effect of ET-1 in normal human arteries [67] and blockade of NO production results in hypertension [68]. This implies that a balance between NO and ET-1 system may play an important role in maintaining vascular homeostasis [69-71]. In the context of vasculopathies, an upregulation of NO production, either by the use of NO donors, eNOS gene transfer or other mechanisms has been demonstrated to inhibit neointima formation and VSMC proliferation in animal models and confer vasculoprotection [72-74]. However, precise molecular events that contribute to this protective response remain poorly defined. Since an exaggerated migration, proliferation and hypertrophy of VSMC play an important role in vascular proliferative diseases, several studies have focussed on investigating the effect of the NO system on the signaling pathways that mediate these responses. In this regard, ET-1-induced phosphorylation of ERK1/2 in pulmonary VSMC of rats was shown to be potently inhibited by the NO donor SNP (sodium nitroprusside) [75]. Interestingly, in these studies, treatment of VSMC with L-NAME (nitro-L-arginine methyl ester), a nonspecific inhibitor of NOS, amplified ET-1-induced ERK1/2 phosphorylation, suggesting that alterations in NO levels can modify the ET-1-induced signaling responses [75]. A similar effect of SNAP (s-nitroso-N-acetylpencillamine), another NO donor, as well as L-NAME, on ET-1-induced ERK1/2 phosphorylation in A10 VSMC has also been reported in studies from our laboratory (Fig. **2A** and **2B**) [76]. Since SNAP raises intracellular cGMP levels *via* sGC in A10 VSMC [65,66], additional studies demonstrated that 8-Br-cGMP (8-bromoguanosine 3’, 5’-cyclic monophosphate), a non-hydro-lyzable analogue of cGMP, mimicked the effect of SNAP and SNP and inhibited ET-1 stimulated ERK1/2 phosphorylation (Fig. **2C**) [76]. Moreover, the ability of ODQ (1*H*-[1,2,4] oxadiazolo[4,3,-a]quino-xalin-1-one), a selective sGC inhibitor, to reverse SNAP-induced attenuation of ERK1/2 phosphorylation (Fig. **2D**) [76] established a role of activated sGC and cGMP in mediating the effects of these NO donors on ET-1-induced ERK signaling. Consistent with these observations, an involvement of cGMP elevation and PKG activation in ET-1-induced ERK phosphorylation in neonatal rat ventricular myocytes have also been reported [5]. In addition to ET-1, angiotensin II, insulin and IGF-1-induced ERK1/2 and other signaling events in cardiac fibroblasts, as well as in VSMC, have been shown to be attenuated by NO donors [77-79]. SNAP treatment was also shown to inhibit ET-1-induced total protein synthesis, an index of hypertrophy, in A10 VSMC (Fig. **3**) as well as in cardiomyocytes [5,76,80].

In the case of A10 VSMC, no attempts were made to directly test the role of cGMP in mediating the antihypertrophic response of SNAP [76], however, in cardiomyocytes this effect appeared to be cGMP-dependent [5,80]. In these studies 8-Br-cGMP mimicked the effect of SNAP on hypertrophic responses [5,80], and pharmacological blockade of PKG by KT5823, a selective PKG inhibitor, reversed the inhibitory effect of SNAP on ET-1-induced phosphorylation of ERK1/2 and c-fos gene expression [5].

**Fig. (4):**
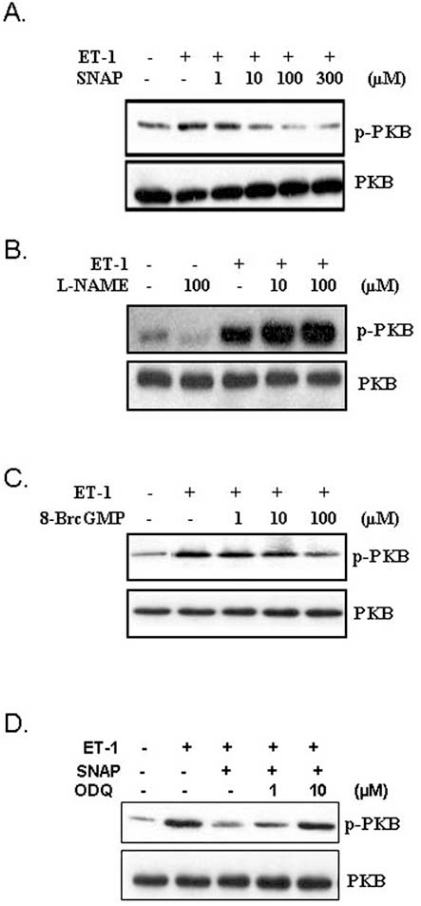
**Modulation of ET-1-induced PKB phosphorylation by NO/cGMP system in A10 VSMC**. **A**.NO donor, SNAP dose-dependently reduced ET-1-induced PKB phosphorylation. **B**.LNAME, NO synthase inhibitor, dose-dependently potentiated ET-1- induced PKB phosphorylation. **C**.cGMP analog, 8-Br-cGMP, mimicked SNAP effect by dose-dependently attenuating ET-1-induced phosphorylation of PKB. **D**.ODQ, an inhibitor of sGC, reversed SNAP-induced attenuation of PKB phosphorylation in response to ET-1. Adapted from [76].

**Fig. (5):**
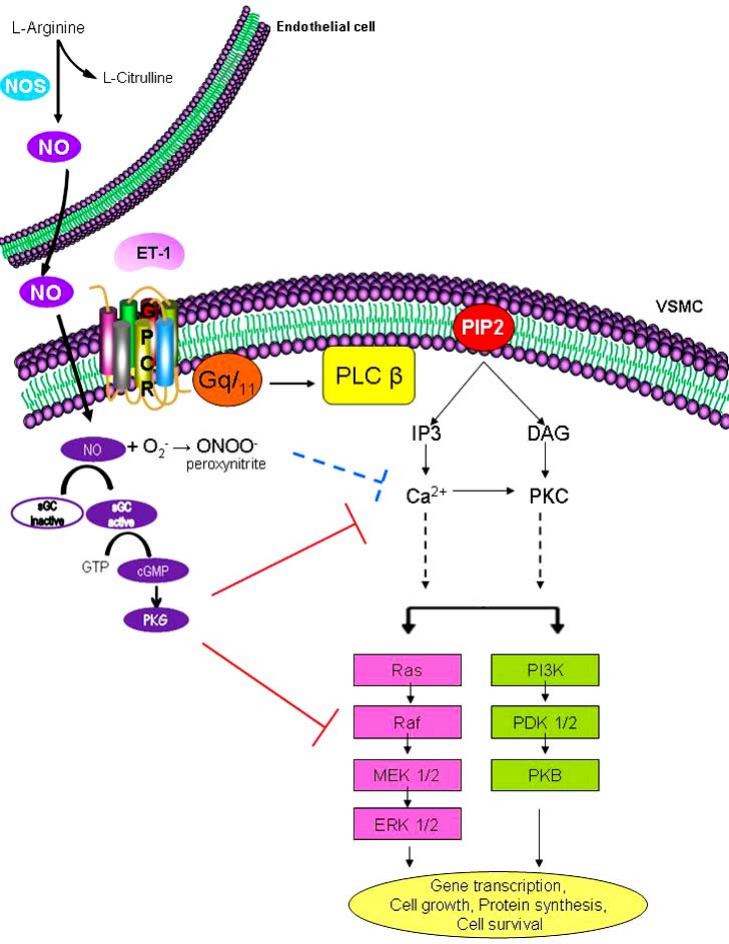
A schematic model summarizing the potential mechanism by which NO system may attenuate ET-1-induced ERK1/2 and PKB signaling in A10 VSMC.

In addition to the ERK pathways, NO has also been shown to attenuate ET-1-induced phosphorylation of PKB in A10 VSMC (Fig. **4A**) [76]. As was the case with ERK pathway, treatment with L-NAME potentiated ET-1-induced phosphorylation of PKB (Fig. **2B**), further suggesting a modulatory role of the NO system on ET-1-induced signaling responses. Moreover, in these studies, 8-Br-cGMP mimicked and ODQ restored the inhibitory effect of SNAP on ET-1-induced phosphorylation of PKB (Figs. **4C** and **4D**) [76]. These studies suggested a role of cGMP-dependent events in modulating NO-induced inhibition of ERK and PKB signaling pathways induced by ET-1. Although not studied in VSMC, ET-1-induced activation of RhoA-ROCK-dependent signaling pathway that participates in cardiac hypertrophy was also reported to be attenuated by SNAP and 8-Br-cGMP [80]. In these studies, ET-1-induced translocation of RhoA and phosphorylation of cofilin-2, a downstream effector of RhoA-ROCK signaling, was significantly inhibited by SNAP as well as 8-Br-cGMP. Consistent with a role of the cGMP system in mediating the antihypertrophic and antiproliferative effects of the NO generating system, multiple studies using isolated cells and animal models have demonstrated an involvement of PKG in this process. For example, expression of a constitutively active PKG was shown to increase the antiproliferative effects of NO in VSMC [81] and to reduce neointimal growth in a model of in-stent restenosis [82]. In addition, high-glucose-induced proliferation and migration of VSMC was also inhibited by overexpression of PKG-1 in VSMC [57]. Despite these observations supporting a role of the cGMP/PKG system, and cGMP-independent events have also been proposed to mediate NO response. For example, a role of cAMP in mediating the growth, inhibitory and signaling responses of NO has been suggested in VSMC [63,65]. Moreover, studies using mouse models lacking PKG-1 have indicated that non-PKG-dependent pathways may also contribute to some of the antihypertrophic and antiproliferative effects of NO [83,84]. In addition, NO-induced peroxynitrite (ONOO^-^) generation has also been proposed to mediate some of the cGMP-independent effects of NO [64,85] (Fig. **5**).

The mechanism by which the NO/cGMP system attenuates ET-1-induced signaling pathways is poorly understood, however, there are reports indicating that NO can inhibit cytoplasmic Ca^2+^ levels [86,87] and, since increased Ca^2+^ is critical to trigger downstream signaling events of ET-1, a decreased Ca^2+^ would turn off the ET-1-induced signaling response (Fig. **5**). Additionally, PKG-induced phosphorylation of c-Raf kinase on serine 43, which results in the uncoupling between Ras and Raf, can block ERK phosphorylation by ET-1 [60] (Fig. **5**). NO generation has also been shown to attenuate IGF-1 and insulin-induced elevation in H_2_O_2_ levels through a cGMP-dependent event in VSMC [79]. ET-1-induced ERK1/2 and PKB signaling is known to require activation of the NADPH-oxidase system, resulting in H_2_O_2_ generation [40], thus, it is possible that NO/cGMP-induced reduction in H_2_O_2_ generation may also contribute to attenuation of ET-1-induced signaling in VSMC.

## CONCLUSION

NO is a well established vasculoprotective agent. Stimulation of sGC leading to enhanced production of cGMP, which in turn activates PKG, appears to be one of the principal pathways involved in mediating the effect of NO. NO has been suggested to antagonize the physiological and pathological effects of growth factors and vasoactive peptides, such as ET-1. NO-induced inhibition of one or more serine/threonine kinases such as ERK1/2 and PKB, implicated in triggering the hypertrophic and hyperproliferative responses in VSMC, may be one of the mechanisms by which this is accomplished. However, the precise molecular events that trigger this effect are poorly understood. Both cGMP-dependent and independent pathways may participate in this process in a context and cell specific fashion. Additional studies using both pharmacological and genetic approaches will help to better enhance our knowledge in this area.

## ACKNOWLEDGEMENTS

This work was supported by funding from the Canadian Institutes of Health Research (CIHR) to AKS. AB was a recipient of a CIHR/RX&D-Canadian Hypertension Society Doctoral Research Award.
